# Getting health services to three million people with TB

**DOI:** 10.7448/IAS.17.1.19079

**Published:** 2014-03-24

**Authors:** Héctor Pérez

**Affiliations:** Infectious Diseases Division, Fernandez Hospital, Buenos Aires, Argentina

Tuberculosis (TB) is the second leading cause of mortality associated with infectious diseases globally and the leading cause of death among people living with HIV. In 2010, there were an estimated nine million people with TB – a third of whom do not have access to TB services – and approximately 1.5 million deaths were attributed to this disease [1]. Moreover, TB is the main opportunistic infection in certain areas, such as Eastern Europe and Central Asia; here, infections are still increasing. Although antiretroviral therapy reduces mortality, there is a need to provide guidelines on the pharmacological interactions between antiviral drugs and drugs used for treating TB.

This year, the STOP TB PARTNERSHIP is focusing on diagnosis, treatment and cure for all [2].

Comparing the pathogenesis of TB with other infections, most infected people can remain asymptomatic for decades. The clinical definition of a latent infection is limited to a positive response to an injection of purified mycobacterial components. The spectrum of active disease is complex. Adult pulmonary TB is considered a public health problem, and extrapulmonary disease occurs in a significant proportion of co-infected patients. Also, paediatric manifestations are difficult to diagnosis. Moreover, many of the 3 million undiagnosed people live in the world's poorest and most vulnerable communities [2, 3].

Treatment is complicated. The combination of drugs requires sequenced administration, unlike other antibacterial treatments that have to be administered for a minimum of six months. The complex interplays between the genetic strain of the bacteria, immune response of the host, and pathogenicity and acquisition of resistance increase the challenges posed by the global TB epidemic and require the development of appropriate approaches for the discovery of drugs and diagnostic techniques applicable in these settings. More research is needed in order to increase our understanding of, and evaluate, the natural history of TB in the context of co-infections, comorbidities and risk behaviours [3].

Microbial resistance has existed since the dawn of the antibiotic era. Multidrug-resistant (MDR) TB requires treatment for up to two years, with success rates of about 65–75%. Extensively drug-resistant (XDR) TB involves additional resistance to two second-line drugs; it is more difficult to treat and may be incurable. One study published in the *New England Journal of Medicine* in 2012 and based on findings from 4000 patients monitored by the Chinese Center for Disease Control and Prevention showed that in newly diagnosed TB cases, 34.2% had some form of drug resistance, 5.7% had MDR TB and 0.5% had XDR TB. Among those previously treated, 54.5% had some form of drug resistance, 25.6% had MDR TB and 2.1% had XDR TB [4].

The sputum smear misses diagnosis in a half of those infected and provides no information about drug resistance. In low-income and middle-income countries, where the global burden is heaviest, sputum, solid culture and chest radiography are scarcely available for diagnosis. However, these tests do not have sufficient sensitivity and specificity, and they are usually not used or available at the periphery of the health system.

The ideal test for TB is a true point-of-care test that enables accurate diagnosis and the detection of drug resistance within the time of clinic consultation.

The biggest challenge today is to progress in the development of new diagnostic assays.

Rapid progress has been made in the development of new diagnostic assays for TB in recent years. New technologies have been developed. The Xpert MTB/RIF assay, which enables simultaneous detection of *Mycobacterium tuberculosis* (MTB) and rifampicin (RIF) resistance, was endorsed by the World Health Organization (WHO) in December 2010. This assay was specifically recommended for use as the initial diagnostic test for suspected drug-resistant or HIV-associated pulmonary TB. Although the development of the Xpert MTB/RIF assay is undoubtedly a landmark, the clinical and programmatic effects and cost-effectiveness remain to be defined [5].

Future advances in molecular diagnosis should build on this success. Despite recent developments, progress in nucleic acid amplification is still weak and should be strengthened.

A rapid, accurate point-of-care diagnostic test that is affordable and can be readily implemented is urgently needed. Investment in the TB diagnostics pipeline should remain a major priority for funders and researchers.

Another objective of the World TB Day is treatment and cure for all. New treatments and regimens for patients with drug-resistant as well as drug-sensitive TB are needed. The long duration of treatment and poor tolerability represent important challenges.

As stated, the incidence of MDR in certain regions, such as eastern Europe, southern Africa and central Asia, is a current problem, as is retreatment as compared with new cases. Shorter regimens are essential. With new drugs for TB, multidrug regimens will require considerations of interactions, pharmacokinetics, toxicity, drug metabolism and the thresholds for the development of resistance. The recognition that patients with TB frequently present with co-infections and comorbidities, such as HIV or other viral infections (e.g., the hepatitis B and hepatitis C viruses), must be assessed at the earliest possible stage in clinical development [3].

There are new drugs in a pipeline that is more robust than ever. Advancing the planning and coordinated assessment of treatment combinations is a high priority. Trials of a novel nitro-dihydro-imidazooxazole (delamanid), together with a background regimen of second-line drugs for MDR TB, resulted in culture conversion at eight weeks, increasing from 30% in placebo versus 45% in the delamanid group. A study of another novel agent, bedaquiline, showed similar results to the delamanid study, although the placebo group showed substantially lower results (9% efficacy). This may be due to the inclusion of more potent fluoroquinolones in the delamanid study [4].

The REMoxTBtrial13 replaced either isoniazid or ethambutol with the fluoroquinolone moxifloxacin (M) in two experimental, four-month regimens (2HRZM/2HRM and 2MRZE/2MR); results are expected shortly. If the findings from the REMoxTB trial are positive, regulatory approval will be sought in 2014 and a national launch could start as early as 2015 [6]. Moxifloxacin, improved the activity of the standard drug regimen when substituted for ethambutol. It’s being studied to shorten the duration of treatment in Remox study.

Finally, eliminating TB and TB/HIV in the 21st century is a priority for various high-level partners. This has been delineated by UNAIDS, world leaders and the United Nations General Assembly, who are committed, by 2015, to [1]:

- Reduce sexual transmission of HIV.
- Halve the rate of HIV infection among persons who inject drugs.
- Eliminate new HIV infections among children.
- Increase the number of persons on life-saving treatment to 15 million.
- Reduce by half the number of TB-related deaths in persons living with HIV.

In summary, World TB Day on 24 March 2014 brought with it the vision and possibility of achieving “a TB test, treatment and cure for all.”
